# Therapeutic Targeting of Cancer: Epigenetic Homeostasis

**DOI:** 10.3389/fonc.2021.747022

**Published:** 2021-10-26

**Authors:** Xiaoyuan Yu, Menglu Li, Chunyan Guo, Yuesheng Wu, Li Zhao, Qinying Shi, Jianbo Song, Bin Song

**Affiliations:** ^1^ Department of Oncology, First Hospital of Shanxi Medical University, Taiyuan, China; ^2^ Shanxi Key Laboratory of Otorhinolaryngology Head and Neck Cancer, First Hospital of Shanxi Medical University, Taiyuan, China; ^3^ Cancer Center, Shanxi Bethune Hospital, Shanxi Academy of Medical Sciences, Tongji Shanxi Hospital, Third Hospital of Shanxi Medical University, Taiyuan, China

**Keywords:** oncobiology, cancer heterogeneity, epigenetics, therapeutic target, transdifferentiation

## Abstract

A large number of studies have revealed that epigenetics plays an important role in cancer development. However, the currently-developed epigenetic drugs cannot achieve a stable curative effect. Thus, it may be necessary to redefine the role of epigenetics in cancer development. It has been shown that embryonic development and tumor development share significant similarities in terms of biological behavior and molecular expression patterns, and epigenetics may be the link between them. Cell differentiation is likely a manifestation of epigenetic homeostasis at the cellular level. In this article, we introduced the importance of epigenetic homeostasis in cancer development and analyzed the shortcomings of current epigenetic treatment regimens. Understanding the dynamic process of epigenetic homeostasis in organ development can help us characterize cancer according to its differentiation stages, explore new targets for cancer treatment, and improve the clinical prognosis of patients with cancer.

## Introduction

As a common disease in multicellular organisms, cancer has always been the focus of scientific research, especially in its pathogenesis (1–4). An increasing number of studies have revealed that epigenetics plays an important role in cancer development. However, existing drugs targeting tumor epigenetics have not achieved stable long-term curative effects. Perhaps we need to rethink the role of epigenetics in cancer development.

In this article, we will refer to existing research to analyze the shortcomings of current epigenetic treatment regimens and review our view: Epigenetic homeostasis refers to the fact that various epigenetic regulatory substances in cells change only in a small range under normal physiological conditions to jointly maintain cell differentiation. Cell differentiation is the manifestation of epigenetic homeostasis at the cellular level, and attention to epigenetic homeostasis may be more important than to level of genome-wide methylation or acetylation. While explaining the important role of epigenetic homeostasis in multicellular organism, the term ‘differentiation lock’ will be used to refer to the composition of epigenetics in cell differentiation. Exploring the dynamic changes in the differentiation lock during organ development may contribute to changing our understanding of cancer and exploring new targets for the epigenetic homeostasis.

## Research Hotspots of Cancer Epigenetics

Several studies have revealed that epigenetics plays an important role in the development of cancer and drug resistance (5), which has stimulated the enthusiasm of researchers (**Table 1**). Current studies mainly focus on histone codes, methyl compounds, and non-coding RNAs (ncRNAs), and related drugs are being developed.

**Table 1:** Associations between epigenetic disorders and cancers.

Cell Type	Epigenetic Abnormalities Characterization	Consequences	References
Breast cancer,	DNA repeat sequence hypermethylation	Reduced stability of the genome	(6, 7)
Lung cancer,			
Liver cancer			
Breast cancer,	Promoter hypomethylation	Protooncogene activated	(8, 9)
Melanoma			
Colorectal cancer,	CpG island hypermethylation	Tumor suppressor genes are inhibited	(10, 11)
Gastric cancer			
Acute myelocytic leukemia	High expression of demethylase FTO	Tumor suppressor genes are inhibited	(12, 13)
Pancreatic cancer,	High expression of demethylase ALKBH5	Promoting self-renewal and proliferation of tumor stem cells	(14, 15)
Liver cancer			
Lung cancer,	High expression of METTL3	Increased growth, survival and invasion of cancer cells	(15)
Liver cancer			
Colorectal cancer,	Loss of H4K16ac / H3K4me3 / H4K20me3,	Transcriptional function was inhibited	(16)
Prostate cancer,	Increase of H3K9me / H3K27me3		
Gastric cancer			
Glioma,	Overexpression of miR-218, miR-21, miR-15b, miR-515-5p	Inhibition of migration, invasion and proliferation of cancer cells	(17, 18)
Breast cancer,			
Lung cancer			
Hepatocellular carcinoma,	Overexpression of miR-125b and miR-346	Promoting cancer cell metastasis and invasion	(19, 20)
Breast cancer,			
Renal cell carcinoma			
Hepatoma	Overexpression of lncTCF7, lnc-β-Catm, lncBRM	Promoting self-renewal of cancer stem cells	(21, 22)
Promyelocytic leukemia,	Overexpression of f-circRNA, circ-Amotl1	Promoting transformation and proliferation of cancer cells	(20, 23, 24)
Breast cancer			

### Histone Code

The nucleosome is the basic chromatin repeating unit, and the core histones that make up the nucleosome are small proteins. Histone modifications mainly include acetylation, methylation, and ubiquitination, and the histone modification state controls whether the transcription complex can come into close proximity with the target gene, affecting its expression activity (25–27). The quantity, position, and type of histone modifications are collectively referred to as histone codes, which play an important role in cell differentiation and maintenance (28–30). Studies have shown that abnormal expression of histone codes is an important feature of cancer tissues and is related to the heterogeneity of cancer cells (31, 32). Studies have shown that abnormal levels of the histone demethylases, KDM6A and KDM6B, are associated with pediatric acute myeloid leukemia (AML) (33). Moreover, modification of the histone proteins H3K9ac, H3K27ac, and H4K16ac plays an important role in the progression and prognosis of head and neck squamous cell carcinoma (HNSCC) (34).

### Methyl Compounds

DNA methylation widely exists in prokaryotes and eukaryotes and is an epigenetic mechanism controlling gene expression (35, 36). Previous studies have revealed epigenetic reprogramming during embryo development (5, 37, 38). With cell differentiation, new methylation patterns are formed to ensure the specific expression of genes in organisms (39, 40). DNA methylation is catalyzed by methyltransferases, including DNMT1, DNMT3A, and DNMT3B (41). Among these enzymes, DNMT1 is responsible for the transmission of methylation patterns during mitosis to prevent passive demethylation. Dysplasia and death have been observed in DNMT1 knockout mice (42). DNMT3A and DNMT3B can methylate unmethylated CpG sites, which is important for embryonic development and tumorigenesis (43, 44). Methylation of cytosine residues leads to gene silencing, which plays a key role in the proper regulation of gene expression, genomic imprinting, X-inactivation, and development. Interestingly, abnormal DNA methylation is often observed in clinical specimens of cancer tissues (45). During tumorigenesis, abnormally high methylation of cytosines in promoter CpG islands, as well as overall gene hypomethylation, lead to genome-wide instability and altered gene expression profiles, including silencing of oncogenes, activation of endogenous retroviruses, and upregulation of tumor antigens and oncogene expression (46, 47). Tumor-specific methylated genes can be detected in circulating tumor cells, blood, urine, and other body fluids and are therefore commonly used in the diagnosis and prognosis of early-stage tumors (48, 49).

### Non-Coding RNA

Non-coding RNAs (ncRNAs), which are not involved in protein-coding, mainly include microRNAs (miRNAs), circular RNAs (circRNAs), ribosomal RNAs (rRNAs), transfer RNAs (tRNAs), small nuclear RNAs (snRNAs), and small nucleolar RNAs (snoRNAs) (50–52). ncRNAs mainly affect gene expression at the transcriptional and translational levels, and play an indispensable role in embryonic development, cell differentiation, damage repair, and regulation of cell function (53–55). Dicer1 is one of the most important enzymes that produce miRNAs. It has been reported that Dicer1-deficient mice have abnormal organ development or face embryonic death, which was attributed to the failure of embryos to correctly process miRNAs (56). Abnormal ncRNAs have been reported to play an important role in tumorigenesis, metastasis, and drug resistance (51, 52, 57). For instance, miRNA-143 regulates a variety of signaling pathways, including WNT/β-catenin, RAS-MAPK, and PI3K/AKT, thereby affecting tumor growth (58). miR-193a-5p promotes tumor cell metastasis by regulating the EMT signaling pathway (59). Furthermore, both germline and somatic mutations in Dicer1 have been identified in diverse types of cancer (60, 61). The errors in ncRNAs are closely related to cancer. However, the exact mechanism is still surprisingly controversial. This may be related to the complexity of the ncRNA regulatory mechanism.

## Current Situation of Epigenetic Drugs

A wide range of therapeutic strategies in cancer treatment are compared to conventional chemotherapeutic agents that target cell proliferation. Currently, epigenetic drugs are being progressively developed and used for cancer treatment (62), such as DNA methyltransferase inhibitors (DNMTi) and histone deacetylase inhibitors (HDACi).

### DNMTi

By inhibiting the activity of DNMTs, the expression of tumor suppressor genes is promoted to inhibit the growth of tumor cells. The main nucleoside and non-nucleoside DNMT inhibitors (DNMTis) include azacitidine (AZA) and decita21bine (63). Azacitidine blocks cytosine methylation by noncompetitive inhibition of DNMT1, resulting in the depletion of methyltransferases and DNA hypomethylation, but it is ineffective for quiescent cells that cannot divide (64). Low-dose azacitidine and decitabin can induce reactivation of the genes that were previously silenced by methylation, thereby inducing the formation of new phenotypes, reducing proliferation, and increasing apoptosis of offspring cells. High-dose drugs have cytotoxic effects and can directly cause tumor cell death (65, 66). Azacitidine and decitabin are approved by the Food and Drug Administration (FDA) as first-line drugs for the treatment of myelodysplastic syndromes (MDS) and leukemia. However, these drugs did not show significant efficacy in solid tumors such as gastrointestinal cancer, lung cancer, breast cancer, and melanoma, and their use was limited due to their side effects and drug instability (67, 68). Dniunaite (69) observed that downregulation of miR-155-5p was significantly correlated with promoter methylation in prostate cancer. DOT1L inhibitors SYC-52221 and EPZ004777 inhibited DNMT3A-mutant cell proliferation, inducing cell cycle arrest and terminal differentiation (70).

### HDACi

HDAC is a highly conserved group of enzymes that removes acetyl groups from the tail of histone lysine. HDAC promotes chromatin closure and inhibits gene transcription by deacetylating histones (71). HDACi is a new antitumor drug that regulates gene expression. It has extensive effects on malignant tumors, including inhibition of cell differentiation, cell cycle growth, and angiogenesis, as well as induction of apoptosis, and immune regulation (72). In animal models, HDAC inhibition was found to inhibit tumor growth and reduce malignant proliferation by downregulating positive cell cycle regulators, such as cell cycle proteins D1, c-Myc, and AKT (73, 74).

Currently, vorinostat and romidepsin are approved for the treatment of skin T-cell lymphoma (75). However, these drugs do not achieve long-term stable efficacy (76–78). This is not only related to low drug stability and high toxicity, but also to abnormal pathway activation. Abnormal activation of the PI3K/AKT, MEK/ERK, and FAK signaling pathways has been observed in the treatment of multiple myeloma with HDACi (79, 80). 5−aza−2’-deoxycytidine promotes migration of acute monocytic leukemia cells *via* activation of the CCL2/CCR2/ERK signaling pathway (81).

Since the drugs target DNA methyltransferase and histone deacetylase, those non-specific alteration of cancer cell methylation and acetylation levels cannot inhibit the development of cancer. In contrast, epigenetics may form complex networks in cells, thereby affecting other genes and signaling pathways, that may be an important reason for the existing epigenetic drug resistance. According to the epigenetic landscape theory (82), it is believed that cell differentiation is a manifestation of epigenetic homeostasis. In other words, targeting epigenetic homeostasis at the molecular level is the future direction of epigenetic treatment for cancer.

## The Normal Differentiation Process

In this section, we describe the normal differentiation process, which helps us to further understand the causes of drug resistance in tumor epigenetics and possible future research directions from the perspective of cell differentiation.

**Figure 1:**
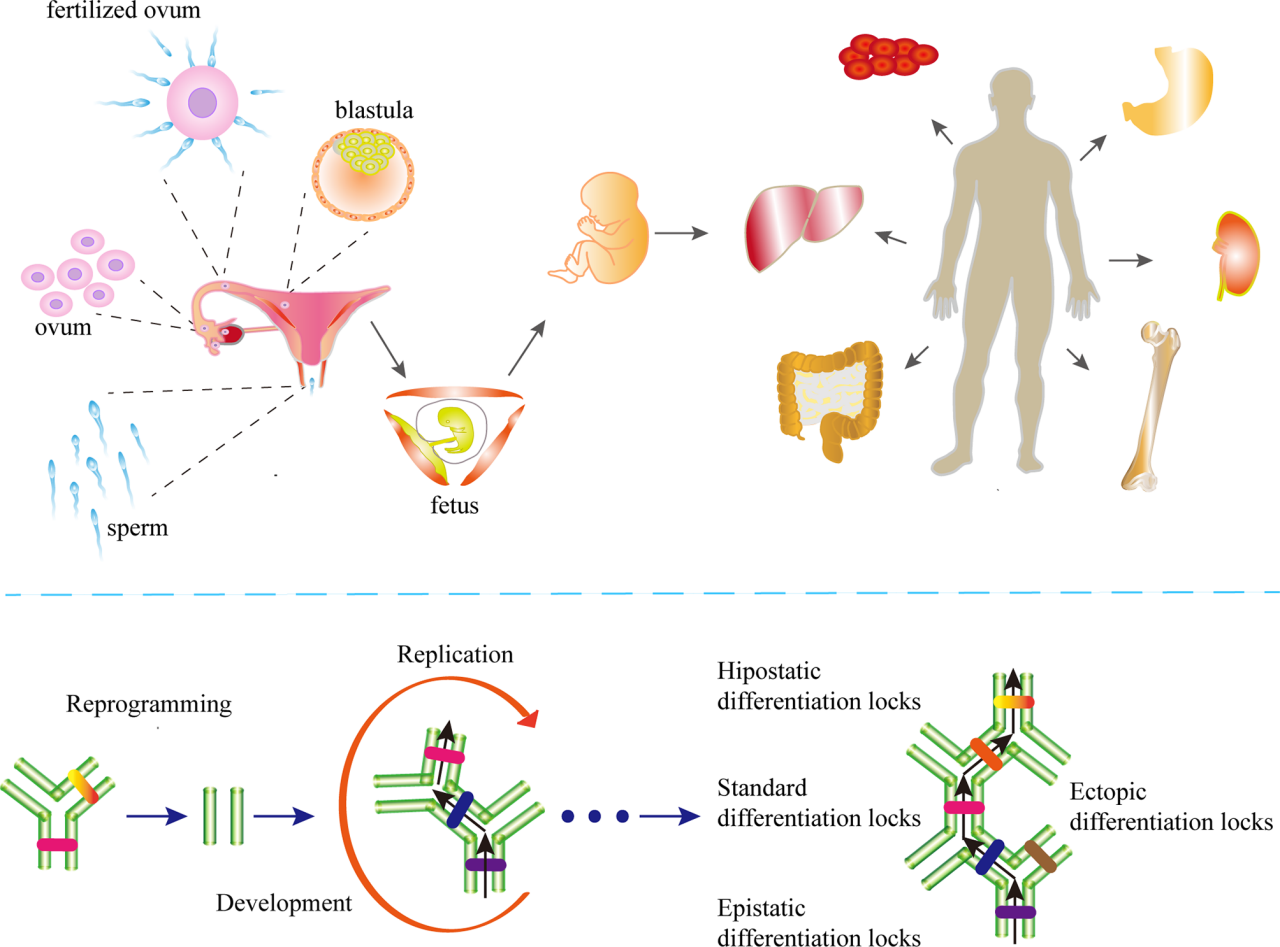
The differentiation locks in cell differentiation. A coordinated gamete and embryo epigenetic reprogramming can eliminate the epigenetic markers carried by their parents, reverse and restore to the state of totipotency, which is the condition of post-fertilization differentiation. Differentiation lock is the composition of epigenetics in cell differentiation The existence of differentiation lock is crucial to maintain the irreversible state of differentiation. The formation of differentiation locks is dependent on intercellular signaling and cell-mesenchymal interactions. The differentiation locks that maintain the state-specific modifications of a cell are called the standard differentiation locks (SDLs), those that maintain the modifications of the previous stage are called the epistatic differentiation locks (EDLs), and the differentiation locks that maintain the modifications of the next stage are called the hypostatic differentiation locks (HDLs). The SDLs may contain parts or all of the EDL modifications. Differentiation locks that do not exist in a cell’s own differentiation path are collectively called ectopic differentiation locks (EcDLs). Differentiation locks replicate, inherit, and develop during the differentiation and division of cells.

The essence of cell differentiation is the expression combination of genes (83). According to classical epigenetic landscape theory, for cells in a certain period, the gene expression profile may maintain dynamic stability (84). This may be attributed to epigenetic homeostasis. Blanca Pijuan-Sala report the transcriptional profiles of single cells from mouse embryos (85), thus portraying the early differentiation trajectory of the mouse embryo and the altered stages of epigenetics in cell differentiation. To characterize epigenetic stage changes, we introduced the concept of differentiation locks and classified differentiation locks into standard differentiation locks (SDLs), epistatic differentiation locks (EDLs) and hypostatic differentiation locks (HDLs) according to the stage cells are in. (**Figure 1**). Changes in epigenetic homeostasis in the cell differentiation pathway have been a hot topic of research. The mouse embryonic hindgut 1-specific genes Trap1a and Rhox5 were also found to be expressed in the ExE endoderm and ExE ectoderm, consistent with the extra-embryonic origin of hindgut 1, suggesting that differentiation locks present different stages in cell differentiation during embryonic development, while the SDLs may inherit some or all of the EDLs (85). Equally important, differentiation lock may be changed, updated during cell division and differentiation to complete the entire differentiation process (86–89). The Human Developmental Cell Atlas (HDCA) is a great human project whose goal is to determine the expression profiles of different human cells and to typify cell differentiation accordingly (90). The theoretical basis of this program is different tissues have distinct differentiation locks, and the same tissues hold similar differentiation locks, thus forming the specificity of tissues and organs and ensuring the normal operation of human functions. The completion of HDCA will help us to deepen our understanding of epigenetic changes during cell differentiation, and will also have a profound impact on the treatment of congenital diseases, cancer and other diseases.

## The Relationship Between the Defective Differentiation Lock and Cancer

With the progress in developmental biology, a large number of studies have revealed that early embryonic development and carcinogenesis have great similarities in biological characteristics such as migration and invasion (91), gene expression and protein spectrum (92), signal pathways (93), cell differentiation (94), and energy metabolism mechanisms (95). Epigenetics has attracted considerable attention as a key cause of these similarities.

Why do mature tissues have biological characteristics similar to those of embryos when transformed into cancer? Daughter cells with defective differentiation lock in their own differentiation path transform into cancer cells during stem cell division. Any factor that can promote the proliferation of stem cells is a promoting factor for cancer. Because the environment forming the ectopic differentiation locks (EcDLs) has changed, cancer cells can no longer differentiate correctly and undergo multidirectional differentiation, resulting in cancer heterogeneity (96).

### The Defective Differentiation Lock Is the Internal Factor of Carcinogenesis

Study have reported the C2H2 zinc finger transcription factor B cell CLL/lymphoma 11A (Bcl11a) is essential for lymphoid development. The deletion of Bcl11a prevents further development of hematopoietic stem cells (HSCs) into lymphocytes (97). Bcl11a^-/-^ HSC alters cell cycle progression. A general upregulation of cell cycle protein genes and a downregulation of the quiescent regulator Cdkn1c (p57) and G2/M markers such as Prc1, Plk1 and Mki67 (Ki-67) of Bcl11a^-/-^ HSC can be observed, and cells eventually appear to proliferate uncontrollably (98, 99).。Similar results can be found in the corresponding studies: DNMTi can inhibit the further differentiation of bone marrow mesenchymal stem cells into osteoblasts and chondrocytes. At the same time, the expression of the anti-senescence genes (TERT, bFGF), and the anti-apoptosis gene (BCL2) was up-regulated and the expression of the apoptotic gene (BAX) was down-regulated (100, 101). The above studies illustrate that cells fail to complete differentiation to form a differentiation lock, i.e., they do not reach epigenetic homeostasis and are transformed into cancer cells. Some mutations in tumor suppressor genes can lead to damaged DNA repair function, such as the BRCA1/2 and P53, and differentiation locks are more likely to be damaged since the gene coding sequences only account for a small proportion of their genome (102, 103). The current research may indirectly confirm our opinion that cancer is a population of cells without epigenetic homeostasis. The non-coding RNA and protein profiles of cancer cells are quite different from those of normal cells, which is also a consequence of the imbalance in epigenetic homeostasis and is often manifested as cell differentiation disorder and cellular dedifferentiation (104, 105).

The consequences of epigenetic modifications may differ according to their position in the differentiation lock. Invisible damage may occur as a result of the defects in EcDL. Cell maturation arrest caused by defective hypostatic differentiation locks (HDLs) results in cancer cell transformation under the continuous stimulation of proliferation signals. Cells with defective standard differentiation locks (SDLs) dedifferentiate and transform into cancer during proliferation, and the invisible damage may become exposed. The invisible damages may explain why the mutation frequency of oncogenes and anti-oncogenes in the human population is much higher than the incidence of cancer (106–109). A possible reason is that tissue-specific alterations in differentiation locks, and defective EcDLs may not impose an effect on the process of tissue carcinogenesis.

Any factor, including physical, chemical, and biological factors, that can damage cells may lead to differentiation lock defects, thereby increasing the incidence of cancer (110–112).

### Stem Cell Division Is a Promoting Factor for Carcinogenesis

In life, a variety of damages and stresses are often met. Mild damage and stress are often dealt with by the asymmetric division of stem cells. When stress exceeds tissue tolerance, stem cells must deal with symmetric divisions (113, 114).

When the cells are asymmetrically divided, the HDL defect (if exists) will lead to the maturation arrest of the daughter cells. These cells cannot complete the next stage of differentiation, and some of the daughter cells die. However, some daughter cells survive and transform into cancer cells under the stimulation of a continuous proliferation signal. When the stem cells are symmetrically divided, the defects of the SDLs are exposed (if exists). As a result, stem cells are dedifferentiated to transform into cancer cells (115). Cell carcinogenesis is a gradual process, and epigenetic homeostasis has a certain tolerance to damage. DNMT1 mainly maintains DNA methylation pattern during DNA replication, ensuring that the pattern is inherited by the offspring. In the early stages, defects in DNMT1 may be related to the accumulation of cell mutations. When the damage exceeds the steady-state tolerance, the cells become cancerous, which may be the internal relationship between aging and cancer.

Therefore, any pressure to stimulate stem cell division can increase the possibility of defective differentiation lock exposure, including injury, infection, and chronic inflammation (116–119).

### Role of Differentiation Lock in Cancer Heterogeneity

As discussed above, the essence of tumor is cells that cannot form epigenetic homeostasis. The correct differentiation process depends on information transmission between cells and the interaction between cells and stroma (120, 121). The environment required for normal development has disappeared, and cancer cells cannot form the correct differentiation lock. Dr. Tushar reported bone marrow microenvironment lead to β-catenin activation and disease progression of MDS (122). Just like without the help of molecular chaperones, peptide chains cannot form proteins properly. Under internal gene-driven and error-induced environments, cancer cells produce heterogeneous daughter cancer cells (123, 124).

Numerous studies comparing gene expression in tumor tissues with paracancerous tissues have found that a large number of genes normally silenced during cell differentiation were activated in cancer. Moreover, these differences in gene expression patterns correlated with the malignancy of the tumor (125, 126). Those studies suggest that epigenetic homeostasis makes a crucial contribution to malignancy.

**Figure 2:**
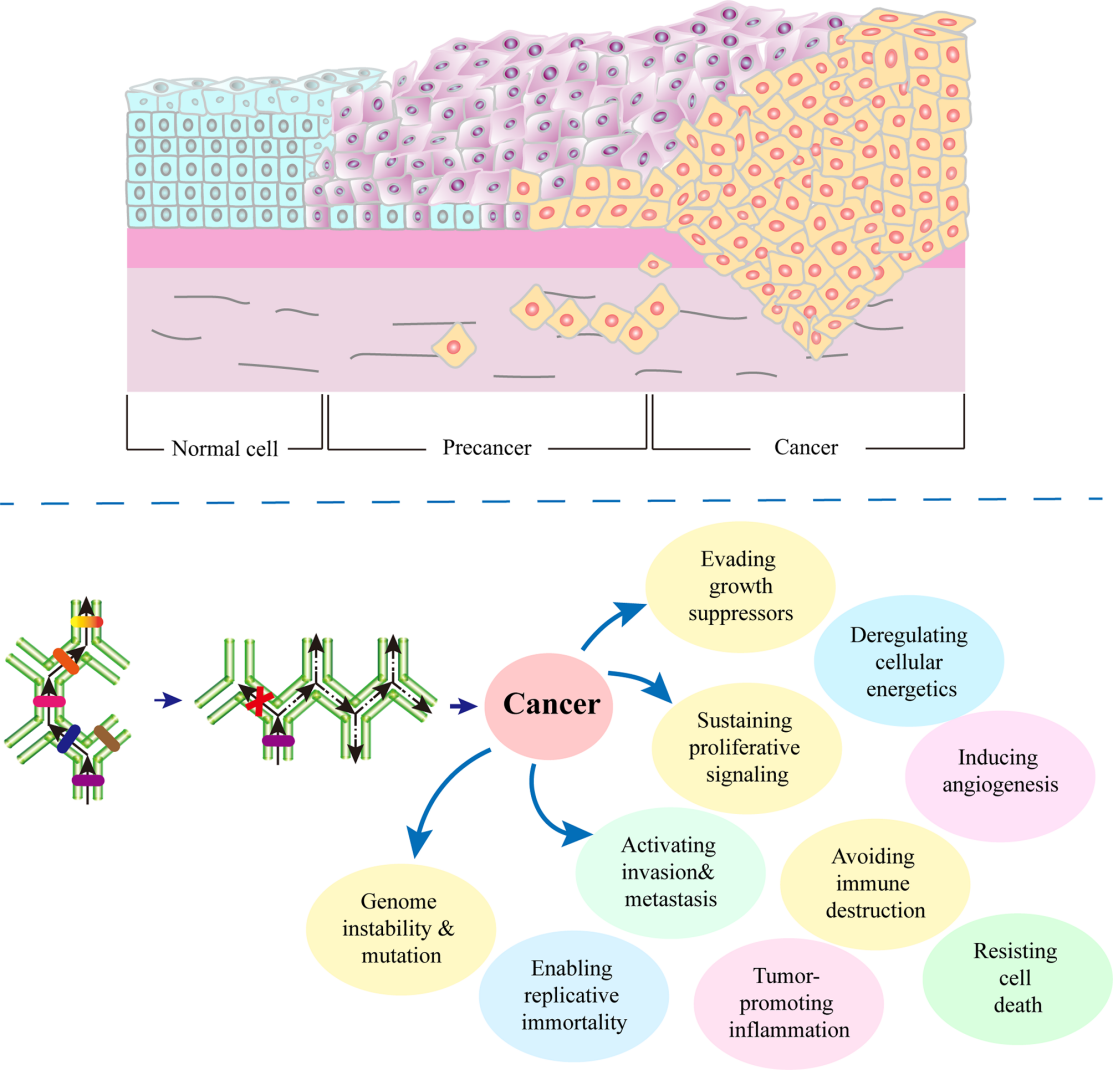
Role of defective differentiation lock in cancer development. A cell can be damaged by physical, chemical, biological and other factors. Depending on the site of damage, there may be different consequences. In the process of cell carcinogenesis, the occurrence of a defective differentiation lock in the differentiation path is the turning point of cell fate. Any factors that promote division, such as chronic inflammation, injury, and infection, will increase the probability of a defective differentiation lock, thereby accelerating the occurrence of cancer. Meanwhile, due to the following two reasons, cancer cells are heterogeneous: 1) the cells cannot complete their differentiation path due to the defects in self-differentiation lock, 2) without correct development environment, ectopic differentiation lock cannot be formed.

The more distinct the defective EDLs from SDLs, the lower the differentiation degree of cells, and the more apparent the characteristics of malignant tumors are (127–129), and vice versa. With the further cancer development, the differentiation locks in the upper layer will change, and the cells will show the characteristics of a lower differentiation stage. At this point, cancer tissues show typical malignant tumor characteristics (130, 131).(**Figure 2**) In the late stage of cancer development, some or all genes that had been closed in the embryonic stages are open.

## Cancer Treatment Strategies for Epigenetic Homeostasis

At present, there are two kinds of tumor treatment strategies for epigenetic homeostasis: 1. Destroy epigenetic homeostasis, thereby inducing apoptosis of tumor cells, strengthening immunogenicity and weakening drug resistance. 2. Reestablish new epigenetic homeostasis to transdifferentiate tumor cells. At present, the two treatment strategies are still in the preclinical stage or clinical trial stage, but they have shown exciting therapeutic prospects.

### Destroying Epigenetic Homeostasis

Epigenetic abnormalities in tumor cells have always been of interest to scientists and physicians. Scholars have found that epigenetically abnormal tumor cells often show apoptosis, which may be a target for tumor therapy. Destruction of epigenetic homeostasis can induce apoptosis of cancer cells. Existing studies have found that cisplatin can not only directly kill cancer by DNA crosslinking, but also induce epigenetic changes of cancer cells and further induce apoptosis of cancer cells (132). Destruction of epigenetic homeostasis may enhance cancer immunogenicity. Researchers have found that inhibition of the histone demethylase LSD1 enhances tumor immunogenicity and T-cell infiltration in tumors. Thus, LSD1 could be used as an anti-tumor immunosuppressant in combination with anti-PD-1 immunotherapy for tumor treatment, with encouraging results in small-scale clinical trials (133, 134). Disruption of epigenetic homeostasis may alter the drug resistance of tumor cells. Methylguanine methyltransferase (MGMT), which repairs temozolomide (TMZ)-induced O6-methylguanine (O6mG) adducts, induces drug resistance in tumor cells. New study has shown reduced production of MGMT in the presence of epigenetic instability, demonstrating that generating epigenetic instability through destruction of epigenetic homeostasis may be a viable strategy to mitigate anticancer drug resistance (135). The above studies are all based on the biological effects generated after the destruction of epigenetic homeostasis to kill tumor cells, which are the current research hotspots.

### Reestablishing Epigenetic Homeostasis

As mentioned above, imbalance of epigenetic homeostasis may be the etiology of tumors. Some scholars believe that by re-establishing epigenetic homeostasis may be a new strategy for cancer treatment. Surprisingly, some studies have reported that breast cancer cells can be terminally differentiated into adipocytes by using the rosiglitazone combined with the trametinib (136, 137). This exciting finding suggests that re-establishing epigenetic homeostasis, i.e., tumor cell transdifferentiation, may be the therapeutic direction for cancer. After transdifferentiation, tumor cells are transformed into new, solid, controlled cells, and patients may achieve durable, stable remission as a result. This fascinating therapeutic prospect has attracted the attention of scientists. However, the lack of understanding of human cell differentiation pathways and differentiation-inducing conditions continues to limit the development of cancer transdifferentiation therapies.

## Discussion and Future Perspectives

Epigenetic changes may occur gradually during cell development. At a specific stage, the composition of epigenetics is stable and maintains the corresponding state of cell differentiation, which is the role of differentiation lock. As discussed above, tumors are cells without epigenetic homeostasis. Existing epigenetic drugs alter the epigenetic status of cancer cells to kill these cells through several pathways. Unfortunately, these drugs have also led to serious adverse effects. In addition, maintaining the low methylation level of cancer cells inevitably leads to the activation of multiple signaling pathways, which is one of the reasons for the consequent emergence of resistance to epigenetic drugs.

With the above discussion, epigenetic homeostasis treatment strategies for tumors may be divided into disruption and reestablishment of epigenetic homeostasis. The biological effects generated by the destruction of epigenetic homeostasis can effectively kill tumors. Reestablishing epigenetic homeostasis may be the focus of future development. Patients achieve long-lasting remission through tumor cell transdifferentiation. To achieve this goal, the following efforts are required: 1) mapping of the differentiation lock changes that arise during cell development to characterize cancer cells, 2) characterization of the induction conditions for differentiation lock to induce cancer cell transdifferentiation. These may require further collaboration between cancer and embryonic development researchers. Understanding the significance of differentiation lock in the organ differentiation process is helpful for developing more effective targeted therapy strategies and implementing individualized treatment for cancer patients by inducing cancer cell transdifferentiation. This measure can be used to improve the prognosis of patients with cancer.

## Author Contributions

All authors have read this manuscript and would like to have it considered exclusively for publication in Frontiers in Oncology.

## Funding

This work was supported by the Natural Science Foundation of China [grant numbers: 81703016] and Hospital Fund of Shanxi Bethune Hospital[grant numbers: 2020ZL02].

## Conflict of Interest

The authors declare that the research was conducted in the absence of any commercial or financial relationships that could be construed as a potential conflict of interest.

## Publisher’s Note

All claims expressed in this article are solely those of the authors and do not necessarily represent those of their affiliated organizations, or those of the publisher, the editors and the reviewers. Any product that may be evaluated in this article, or claim that may be made by its manufacturer, is not guaranteed or endorsed by the publisher.

## References

[1] Muller AWJ. Cancer is an Adaptation That Selects in Animals Against Energy Dissipation. Med Hypotheses (2017) 104:104–15. doi: 10.1016/j.mehy.2017.05.030 28673566

[2] Montalban-Arques A Scharl M. Intestinal Microbiota and Colorectal Carcinoma: Implications for Pathogenesis. Diagn Ther EBioMed (2019) 48:648–55. doi: 10.1016/j.ebiom.2019.09.050 PMC6838386 31631043

[3] Curtius K Wright NA Graham TA. An Evolutionary Perspective on Field Cancerization, Nature Reviews. Cancer (2018) 18:19–32. doi: 10.1038/nrc.2017.102 29217838

[4] DeBerardinis RJ Chandel NS. Fundamentals of Cancer Metabolism. Sci Adv (2016) 2:e1600200. doi: 10.1126/sciadv.1600200 27386546 PMC4928883

[5] Ming H Sun J Pasquariello R Gatenby L Herrick JR Yuan Y. The Landscape of Accessible Chromatin in Bovine Oocytes and Early Embryos. Epigenetics (2021) 16:300–12. doi: 10.1080/15592294.2020.1795602 PMC7901547 32663104

[6] Ponomaryova AA Rykova EY Gervas PA Cherdyntseva NV Mamedov IZ Azhikina TL. Aberrant Methylation of LINE-1 Transposable Elements: A Search for Cancer Biomarkers. Cells (2020) 9. doi: 10.3390/cells9092017 PMC7563416 32887319

[7] Rodic N. LINE-1 Activity and Regulation in Cancer. Front Biosci (Landmark edition) (2018) 23:1680–6. doi: 10.2741/4666 29293456

[8] Johnstone SE Reyes A Qi Y Adriaens C Hegazi E Pelka K. Large-Scale Topological Changes Restrain Malignant Progression in Colorectal Cancer. Cell (2020) 182:1474–89.e1423. doi: 10.1016/j.cell.2020.07.030 32841603 PMC7575124

[9] Holm K Staaf J Lauss M Aine M Lindgren D Bendahl PO. An Integrated Genomics Analysis of Epigenetic Subtypes in Human Breast Tumors Links DNA Methylation Patterns to Chromatin States in Normal Mammary Cells. Breast Cancer Res BCR (2016) 18:27. doi: 10.1186/s13058-016-0685-5 26923702 PMC4770527

[10] Biswas S Rao CM. Epigenetics in Cancer: Fundamentals and Beyond. Pharmacol Ther (2017) 173:118–34. doi: 10.1016/j.pharmthera.2017.02.011 28188812

[11] Wu HJ Chu PY. Epigenetic Regulation of Breast Cancer Stem Cells Contributing to Carcinogenesis and Therapeutic Implications. Int J Mol Sci (2021) 22. doi: 10.3390/ijms22158113 PMC8348144 34360879

[12] Sun K Du Y Hou Y Zhao M Li J Du Y. Saikosaponin D Exhibits Anti-Leukemic Activity by Targeting FTO/M(6)A Signaling. Theranostics (2021) 11:5831–46. doi: 10.7150/thno.55574 PMC8058711 33897884

[13] Huang Y Su R Sheng Y Dong L Dong Z Xu H. Small-Molecule Targeting of Oncogenic FTO Demethylase in Acute Myeloid Leukemia. Cancer Cell (2019) 35:677–91.e610. doi: 10.1016/j.ccell.2019.03.006 30991027 PMC6812656

[14] Guo X Li K Jiang W Hu Y Xiao W Huang Y. RNA Demethylase ALKBH5 Prevents Pancreatic Cancer Progression by Posttranscriptional Activation of PER1 in an M6a-YTHDF2-Dependent Manner. Mol Cancer (2020) 19:91. doi: 10.1186/s12943-020-01158-w 32429928 PMC7236181

[15] Chen Y Zhao Y Chen J Peng C Zhang Y Tong R. ALKBH5 Suppresses Malignancy of Hepatocellular Carcinoma via M(6)A-Guided Epigenetic Inhibition of LYPD1. Mol Cancer (2020) 19:123. doi: 10.1186/s12943-020-01239-w 32772918 PMC7416417

[16] Zhuang J Huo Q Yang F Xie N. Perspectives on the Role of Histone Modification in Breast Cancer Progression and the Advanced Technological Tools to Study Epigenetic Determinants of Metastasis. Front Genet (2020) 11:603552. doi: 10.3389/fgene.2020.603552 33193750 PMC7658393

[17] Regazzo G Terrenato I Spagnuolo M Carosi M Cognetti G Cicchillitti L. A Restricted Signature of Serum miRNAs Distinguishes Glioblastoma From Lower Grade Gliomas. J Exp Clin Cancer Res CR (2016) 35:124. doi: 10.1186/s13046-016-0393-0 27476114 PMC4967504

[18] Kan LK Drummond K Hunn M Williams D O'Brien TJ Monif M. Potential Biomarkers and Challenges in Glioma Diagnosis, Therapy and Prognosis. BMJ Neurol Open (2020) 2:e000069. doi: 10.1136/bmjno-2020-000069 PMC7871709 33681797

[19] Wang Y Zeng G Jiang Y. The Emerging Roles of miR-125b in Cancers. Cancer Manage Res (2020) 12:1079–88. doi: 10.2147/cmar.S232388 PMC7024862 32104088

[20] Su ZH Liao HH Lu KE Chi Z Qiu ZQ Jiang JM. Hypoxia-Responsive miR-346 Promotes Proliferation, Migration, and Invasion of Renal Cell Carcinoma Cells via Targeting NDRG2. Neoplasma (2020) 67:1002–11. doi: 10.4149/neo_2020_190917N915 32453597

[21] Zhu P Wang Y Huang G Ye B Liu B Wu J. Lnc-β-Catm Elicits EZH2-Dependent β-Catenin Stabilization and Sustains Liver CSC Self-Renewal. Nat Struct Mol Biol (2016) 23:631–9. doi: 10.1038/nsmb.3235 27239797

[22] Zhu P Wang Y Wu J Huang G Liu B Ye B. LncBRM Initiates YAP1 Signalling Activation to Drive Self-Renewal of Liver Cancer Stem Cells. Nat Commun (2016) 7:13608. doi: 10.1038/ncomms13608 27905400 PMC5146280

[23] Yang F Hu A Guo Y Wang J Li D Wang X. P113 Isoform Encoded by CUX1 Circular RNA Drives Tumor Progression via Facilitating ZRF1/BRD4 Transactivation. Mol Cancer (2021) 20:123. doi: 10.1186/s12943-021-01421-8 34579723 PMC8474885

[24] Sun K Wang D Yang BB Ma J. The Emerging Functions of Circular RNAs in Bladder Cancer. Cancers (2021) 13. doi: 10.3390/cancers13184618 PMC8464819 34572845

[25] Wang W Meng ZQ Shi FX. Modification and Biological Role of Histone. Yi Chuan = Hereditas (2012) 34:810–8. doi: 10.3724/sp.j.1005.2012.00810 22805206

[26] Pradeepa MM. Causal Role of Histone Acetylations in Enhancer Function. Transcription (2017) 8:40–7. doi: 10.1080/21541264.2016.1253529 PMC5279748 27792455

[27] Schneider J Shilatifard A. Histone Demethylation by Hydroxylation: Chemistry in Action. ACS Chem Biol (2006) 1:75–81. doi: 10.1021/cb600030b 17163647

[28] Orsi GA Couble P Loppin B. Epigenetic and Replacement Roles of Histone Variant H3.3 in Reproduction and Development. Int J Dev Biol (2009) 53:231–43. doi: 10.1387/ijdb.082653go 19412883

[29] Villaseñor R Baubec T. Regulatory Mechanisms Governing Chromatin Organization and Function. Curr Opin Cell Biol (2020) 70:10–7. doi: 10.1016/j.ceb.2020.10.015 33276273

[30] Fyodorov DV Zhou BR Skoultchi AI Bai Y. Emerging Roles of Linker Histones in Regulating Chromatin Structure and Function, Nature Reviews. Mol Cell Biol (2018) 19:192–206. doi: 10.1038/nrm.2017.94 PMC5897046 29018282

[31] Scaffidi P. Histone H1 Alterations in Cancer. Biochim Biophys Acta (2016) 1859:533–9. doi: 10.1016/j.bbagrm.2015.09.008 26386351

[32] Füllgrabe J Kavanagh E Joseph B. Histone Onco-Modifications. Oncogene (2011) 30:3391–403. doi: 10.1038/onc.2011.121 21516126

[33] Jones L McCarthy P Bond J. Epigenetics of Paediatric Acute Myeloid Leukaemia. Br J Haematol (2020) 188:63–76. doi: 10.1111/bjh.16361 31804725

[34] Gaździcka J Gołąbek K Strzelczyk JK Ostrowska Z. Epigenetic Modifications in Head and Neck Cancer. Biochem Genet (2020) 58:213–44. doi: 10.1007/s10528-019-09941-1 PMC7113219 31712935

[35] Bartels A Han Q Nair P Stacey L Gaynier H Mosley M. Dynamic DNA Methylation in Plant Growth and Development. Int J Mol Sci (2018) 19. doi: 10.3390/ijms19072144 PMC6073778 30041459

[36] Law PP Holland ML. DNA Methylation at the Crossroads of Gene and Environment Interactions. Essays Biochem (2019) 63:717–26. doi: 10.1042/ebc20190031 PMC6923319 31782496

[37] Kalkan T Olova N Roode M Mulas C Lee HJ Nett I. Tracking the Embryonic Stem Cell Transition From Ground State Pluripotency. Dev (Cambridge England) (2017) 144:1221–34. doi: 10.1242/dev.142711 PMC5399622 28174249

[38] Iurlaro M von Meyenn F Reik W. DNA Methylation Homeostasis in Human and Mouse Development. Curr Opin Genet Dev (2017) 43:101–9. doi: 10.1016/j.gde.2017.02.003 28260631

[39] Canon E Jouneau L Blachère T Peynot N Daniel N Boulanger L. Progressive Methylation of POU5F1 Regulatory Regions During Blastocyst Development. Reprod (Cambridge England) (2018) 156:145–61. doi: 10.1530/rep-17-0689 29866767

[40] Ikegami K Ohgane J Tanaka S Yagi S Shiota K. Interplay Between DNA Methylation, Histone Modification and Chromatin Remodeling in Stem Cells and During Development. Int J Dev Biol (2009) 53:203–14. doi: 10.1387/ijdb.082741ki 19412882

[41] Okano M Bell DW Haber DA Li E. DNA Methyltransferases Dnmt3a and Dnmt3b are Essential for *De Novo* Methylation and Mammalian Development. Cell (1999) 99:247–57. doi: 10.1016/s0092-8674(00)81656-6 10555141

[42] Kaji K Factor VM Andersen JB Durkin ME Tomokuni A Marquardt JU. DNMT1 Is a Required Genomic Regulator for Murine Liver Histogenesis and Regeneration. Hepatol (Baltimore Md.) (2016) 64:582–98. doi: 10.1002/hep.28563 PMC5841553 26999257

[43] Deevy O Bracken AP. PRC2 Functions in Development and Congenital Disorders. Dev (Cambridge England) (2019) 146. doi: 10.1242/dev.181354 PMC6803372 31575610

[44] Wong KK Lawrie CH Green TM. Oncogenic Roles and Inhibitors of DNMT1, DNMT3A, and DNMT3B in Acute Myeloid Leukaemia. biomark Insights (2019) 14:1177271919846454. doi: 10.1177/1177271919846454 31105426 PMC6509988

[45] Saghafinia S Mina M Riggi N Hanahan D Ciriello G. Pan-Cancer Landscape of Aberrant DNA Methylation Across Human Tumors. Cell Rep (2018) 25:1066–80.e1068. doi: 10.1016/j.celrep.2018.09.082 30355485

[46] Liang G Weisenberger DJ. DNA Methylation Aberrancies as a Guide for Surveillance and Treatment of Human Cancers. Epigenetics (2017) 12:416–32. doi: 10.1080/15592294.2017.1311434 PMC5501209 28358281

[47] Casalino L Verde P. Multifaceted Roles of DNA Methylation in Neoplastic Transformation, From Tumor Suppressors to EMT and Metastasis. Genes (2020) 11. doi: 10.3390/genes11080922 PMC7463745 32806509

[48] Benezeder T Tiran V Treitler AAN Suppan C Rossmann C Stoeger H. Multigene Methylation Analysis of Enriched Circulating Tumor Cells Associates With Poor Progression-Free Survival in Metastatic Breast Cancer Patients. Oncotarget (2017) 8:92483–96. doi: 10.18632/oncotarget.21426 PMC5696198 29190932

[49] Xu W Lu J Zhao Q Wu J Sun J Han B. Genome-Wide Plasma Cell-Free DNA Methylation Profiling Identifies Potential Biomarkers for Lung Cancer. Dis Markers (2019) 2019:4108474. doi: 10.1155/2019/4108474 30867848 PMC6379867

[50] Panni S Lovering RC Porras P Orchard S. Non-Coding RNA Regulatory Networks, Biochimica Et Biophysica Acta. Gene Regul Mech (2020) 1863:194417. doi: 10.1016/j.bbagrm.2019.194417 31493559

[51] Anastasiadou E Jacob LS Slack FJ. Non-Coding RNA Networks in Cancer, Nature Reviews. Cancer (2018) 18:5–18. doi: 10.1038/nrc.2017.99 PMC6337726 29170536

[52] Romano G Veneziano D Acunzo M Croce CM. Small non-Coding RNA and Cancer. Carcinogenesis (2017) 38:485–91. doi: 10.1093/carcin/bgx026 PMC6248440 28449079

[53] Hombach S Kretz M. Non-Coding RNAs: Classification, Biology and Functioning. Adv Exp Med Biol (2016) 937:3–17. doi: 10.1007/978-3-319-42059-2_1 27573892

[54] Mattick JS Makunin IV. Non-Coding RNA. Hum Mol Genet (2006) 15 Spec No 1:R17–29. doi: 10.1093/hmg/ddl046 16651366

[55] Fu Q Liu CJ Zhai ZS Zhang X Qin T Zhang HW. Single-Cell Non-Coding RNA in Embryonic Development. Adv Exp Med Biol (2018) 1068:19–32. doi: 10.1007/978-981-13-0502-3_3 29943293

[56] Zehir A Hua LL Maska EL Morikawa Y Cserjesi P. Dicer is Required for Survival of Differentiating Neural Crest Cells. Dev Biol (2010) 340:459–67. doi: 10.1016/j.ydbio.2010.01.039 PMC2878775 20144605

[57] Chi Y Wang D Wang J Yu W Yang J. Long Non-Coding RNA in the Pathogenesis of Cancers. Cells (2019) 8. doi: 10.3390/cells8091015 PMC6770362 31480503

[58] Tokumaru Y Takabe K Yoshida K Akao Y. Effects of MIR143 on Rat Sarcoma Signaling Networks in Solid Tumors: A Brief Overview. Cancer Sci (2020) 111:1076–83. doi: 10.1111/cas.14357 PMC7156858 32077199

[59] Shirafkan N Shomali N Kazemi T Shanehbandi D Ghasabi M Baghbani E. microRNA-193a-5p Inhibits Migration of Human HT-29 Colon Cancer Cells via Suppression of Metastasis Pathway. J Cell Biochem (2018). doi: 10.1002/jcb.28164 30506718

[60] Foulkes WD Priest JR Duchaine TF. DICER1: Mutations, microRNAs and Mechanisms, Nature Reviews. Cancer (2014) 14:662–72. doi: 10.1038/nrc3802 25176334

[61] Robertson JC Jorcyk CL Oxford JT. DICER1 Syndrome: DICER1 Mutations in Rare Cancers. Cancers (2018) 10. doi: 10.3390/cancers10050143 PMC5977116 29762508

[62] Miranda Furtado CL Dos Santos Luciano MC Silva Santos RD Furtado GP Moraes MO Pessoa C. Epidrugs: Targeting Epigenetic Marks in Cancer Treatment. Epigenetics (2019) 14:1164–76. doi: 10.1080/15592294.2019.1640546 PMC6791710 31282279

[63] Zhou Z Li HQ Liu F. DNA Methyltransferase Inhibitors and Their Therapeutic Potential. Curr Top Med Chem (2018) 18:2448–57. doi: 10.2174/1568026619666181120150122 30465505

[64] Hagemann S Heil O Lyko F Brueckner B. Azacytidine and Decitabine Induce Gene-Specific and non-Random DNA Demethylation in Human Cancer Cell Lines. PloS One (2011) 6:e17388. doi: 10.1371/journal.pone.0017388 21408221 PMC3049766

[65] Seelan RS Mukhopadhyay P Pisano MM Greene RM. Effects of 5-Aza-2'-Deoxycytidine (Decitabine) on Gene Expression. Drug Metab Rev (2018) 50:193–207. doi: 10.1080/03602532.2018.1437446 29455551

[66] Moro H Hattori N Nakamura Y Kimura K Imai T Maeda M. Epigenetic Priming Sensitizes Gastric Cancer Cells to Irinotecan and Cisplatin by Restoring Multiple Pathways. Gastric Cancer Off J Int Gastric Cancer Assoc Jpn Gastric Cancer Assoc (2020) 23:105–15. doi: 10.1007/s10120-019-01010-1 31555951

[67] Gros C Fahy J Halby L Dufau I Erdmann A Gregoire JM. DNA Methylation Inhibitors in Cancer: Recent and Future Approaches. Biochimie (2012) 94:2280–96. doi: 10.1016/j.biochi.2012.07.025 22967704

[68] Lopez M Halby L Arimondo PB. DNA Methyltransferase Inhibitors: Development and Applications. Adv Exp Med Biol (2016) 945:431–73. doi: 10.1007/978-3-319-43624-1_16 27826847

[69] Daniunaite K Dubikaityte M Gibas P Bakavicius A Rimantas Lazutka J Ulys A. Clinical Significance of miRNA Host Gene Promoter Methylation in Prostate Cancer. Hum Mol Genet (2017) 26:2451–61. doi: 10.1093/hmg/ddx138 28398479

[70] Rau RE Rodriguez BA Luo M Jeong M Rosen A Rogers JH. DOT1L as a Therapeutic Target for the Treatment of DNMT3A-Mutant Acute Myeloid Leukemia. Blood (2016) 128:971–81. doi: 10.1182/blood-2015-11-684225 PMC4990856 27335278

[71] Wang P Zhao H Ren F Zhao Q Shi R Liu X. Research Progress of Epigenetics in Pathogenesis and Treatment of Malignant Tumors. Zhongguo Fei Ai Za Zhi = Chin J Lung Cancer (2020) 23:91–100. doi: 10.3779/j.issn.1009-3419.2020.02.04 PMC7049791 32093453

[72] Milazzo G Mercatelli D Di Muzio G Triboli L De Rosa P Perini G. Histone Deacetylases (HDACs): Evolution, Specificity, Role in Transcriptional Complexes, and Pharmacological Actionability. Genes (2020) 11. doi: 10.3390/genes11050556 PMC7288346 32429325

[73] Manzotti G Ciarrocchi A Sancisi V. Inhibition of BET Proteins and Histone Deacetylase (HDACs): Crossing Roads in Cancer Therapy. Cancers (2019) 11. doi: 10.3390/cancers11030304 PMC6468908 30841549

[74] Lin YH Tsui KH Chang KS Hou CP Feng TH Juang HH. Maspin is a PTEN-Upregulated and P53-Upregulated Tumor Suppressor Gene and Acts as an HDAC1 Inhibitor in Human Bladder Cancer. Cancers (2019) 12. doi: 10.3390/cancers12010010 PMC7016534 31861435

[75] Kim YH Bagot M Pinter-Brown L Rook AH Porcu P Horwitz SM. Mogamulizumab Versus Vorinostat in Previously Treated Cutaneous T-Cell Lymphoma (MAVORIC): An International, Open-Label, Randomised, Controlled Phase 3 Trial, The Lancet. Oncology (2018) 19:1192–204. doi: 10.1016/s1470-2045(18)30379-6 30100375

[76] Myasoedova VA Sukhorukov V Grechko AV Zhang D Romanenko E Orekhov V. Inhibitors of DNA Methylation and Histone Deacetylation as Epigenetically Active Drugs for Anticancer Therapy. Curr Pharm Des (2019) 25:635–41. doi: 10.2174/1381612825666190405144026 30950345

[77] Morel D Jeffery D Aspeslagh S Almouzni G Postel-Vinay S. Combining Epigenetic Drugs With Other Therapies for Solid Tumours - Past Lessons and Future Promise, Nature Reviews. Clin Oncol (2020) 17:91–107. doi: 10.1038/s41571-019-0267-4 31570827

[78] Ganesan A Arimondo PB Rots MG Jeronimo C Berdasco M. The Timeline of Epigenetic Drug Discovery: From Reality to Dreams. Clin Epigenet (2019) 11:174. doi: 10.1186/s13148-019-0776-0 PMC6888921 31791394

[79] Kulka LAM Fangmann PV Panfilova D Olzscha H. Impact of HDAC Inhibitors on Protein Quality Control Systems: Consequences for Precision Medicine in Malignant Disease. Front Cell Dev Biol (2020) 8:425. doi: 10.3389/fcell.2020.00425 32582706 PMC7291789

[80] Pinto V Bergantim R Caires HR Seca H Guimarães JE Vasconcelos MH. Multiple Myeloma: Available Therapies and Causes of Drug Resistance. Cancers (2020) 12. doi: 10.3390/cancers12020407 PMC7072128 32050631

[81] Xiao X Xu Q Sun Y Lu Z Li R Wang X. 5−Aza−2'−Deoxycytidine Promotes Migration of Acute Monocytic Leukemia Cells *via* Activation of CCL2−CCR2−ERK Signaling Pathway. Mol Med Rep (2017) 16:1417–24. doi: 10.3892/mmr.2017.6737 28627644

[82] Dambacher S de Almeida GP Schotta G. Dynamic Changes of the Epigenetic Landscape During Cellular Differentiation. Epigenomics (2013) 5:701–13. doi: 10.2217/epi.13.67 24283883

[83] Yadav T Quivy JP Almouzni G. Chromatin Plasticity: A Versatile Landscape That Underlies Cell Fate and Identity. Science (2018) 361:1332–6. doi: 10.1126/science.aat8950 30262494

[84] Williams BP Gehring M. Principles of Epigenetic Homeostasis Shared Between Flowering Plants and Mammals. Trends Genet TIG (2020) 36:751–63. doi: 10.1016/j.tig.2020.06.019 32711945

[85] Pijuan-Sala B Griffiths JA Guibentif C Hiscock TW Jawaid W Calero-Nieto FJ. A Single-Cell Molecular Map of Mouse Gastrulation and Early Organogenesis. Nature (2019) 566:490–5. doi: 10.1038/s41586-019-0933-9 PMC6522369 30787436

[86] Dudka D Meraldi P. Symmetry Does Not Come for Free: Cellular Mechanisms to Achieve a Symmetric Cell Division. Results Probl Cell Differ (2017) 61:301–21. doi: 10.1007/978-3-319-53150-2_14 28409311

[87] Escobar TM Oksuz O Saldaña-Meyer R Descostes N Bonasio R Reinberg D. Active and Repressed Chromatin Domains Exhibit Distinct Nucleosome Segregation During DNA Replication. Cell (2019) 179:953–63.e911. doi: 10.1016/j.cell.2019.10.009 31675501 PMC6917041

[88] Sunchu B Cabernard C. Principles and Mechanisms of Asymmetric Cell Division. Dev (Cambridge England) (2020) 147. doi: 10.1242/dev.167650 PMC7338270 32601056

[89] Corpet A Almouzni G. Making Copies of Chromatin: The Challenge of Nucleosomal Organization and Epigenetic Information. Trends Cell Biol (2009) 19:29–41. doi: 10.1016/j.tcb.2008.10.002 19027300

[90] Haniffa M Taylor D Linnarsson S Aronow BJ Bader GD Barker RA. A Roadmap for the Human Developmental Cell Atlas. Nature (2021) 597:196–205. doi: 10.1038/s41586-021-03620-1 34497388 PMC10337595

[91] Vladar EK Königshoff M. Noncanonical Wnt Planar Cell Polarity Signaling in Lung Development and Disease. Biochem Soc Trans (2020) 48:231–43. doi: 10.1042/bst20190597 PMC8969929 32096543

[92] Ma Y Zhang P Wang F Liu W Yang J Qin H. An Integrated Proteomics and Metabolomics Approach for Defining Oncofetal Biomarkers in the Colorectal Cancer. Ann Surg (2012) 255:720–30. doi: 10.1097/SLA.0b013e31824a9a8b 22395091

[93] Alissafi T Hatzioannou A Legaki AI Varveri A Verginis P. Balancing Cancer Immunotherapy and Immune-Related Adverse Events: The Emerging Role of Regulatory T Cells. J Autoimmun (2019) 104:102310. doi: 10.1016/j.jaut.2019.102310 31421963

[94] Zhou W Wang G Guo S. Regulation of Angiogenesis *via* Notch Signaling in Breast Cancer and Cancer Stem Cells. Biochim Biophys Acta (2013) 1836:304–20. doi: 10.1016/j.bbcan.2013.10.003 PMC7983532 24183943

[95] Turdo A Porcelli G D'Accardo C Franco SD Verona F Forte S. Metabolic Escape Routes of Cancer Stem Cells and Therapeutic Opportunities. Cancers (2020) 12. doi: 10.3390/cancers12061436 PMC7352619 32486505

[96] Hass R von der Ohe J Ungefroren H. Impact of the Tumor Microenvironment on Tumor Heterogeneity and Consequences for Cancer Cell Plasticity and Stemness. Cancers (2020) 12. doi: 10.3390/cancers12123716 PMC7764513 33322354

[97] Yu Y Wang J Khaled W Burke S Li P Chen X. Bcl11a is Essential for Lymphoid Development and Negatively Regulates P53. J Exp Med (2012) 209:2467–83. doi: 10.1084/jem.20121846 PMC3526365 23230003

[98] Tsang JC Yu Y Burke S Buettner F Wang C Kolodziejczyk AA. Single-Cell Transcriptomic Reconstruction Reveals Cell Cycle and Multi-Lineage Differentiation Defects in Bcl11a-Deficient Hematopoietic Stem Cells. Genome Biol (2015) 16:178. doi: 10.1186/s13059-015-0739-5 26387834 PMC4576406

[99] Santos A Wernersson R Jensen LJ. Cyclebase 3.0: A Multi-Organism Database on Cell-Cycle Regulation and Phenotypes. Nucleic Acids Res (2015) 43:D1140–4. doi: 10.1093/nar/gku1092 PMC4383920 25378319

[100] Nomura Y Hara ES Yoshioka Y Nguyen HT Nosho S Komori T. DNA Methylation-Based Regulation of Human Bone Marrow-Derived Mesenchymal Stem/Progenitor Cell Chondrogenic Differentiation. Cells Tissues Organs (2019) 207:115–26. doi: 10.1159/000502885 31574516

[101] Li Q Zhai Y Man X Zhang S An X. Inhibition of DNA Methyltransferase by RG108 Promotes Pluripotency-Related Character of Porcine Bone Marrow Mesenchymal Stem Cells. Cell Reprogram (2020) 22:82–9. doi: 10.1089/cell.2019.0060 32125888

[102] Kanapathipillai M. Treating P53 Mutant Aggregation-Associated Cancer. Cancers (2018) 10. doi: 10.3390/cancers10060154 PMC6025594 29789497

[103] Gumaste PV Penn LA Cymerman RM Kirchhoff T Polsky D McLellan B. Skin Cancer Risk in BRCA1/2 Mutation Carriers. Br J Dermatol (2015) 172:1498–506. doi: 10.1111/bjd.13626 PMC5785081 25524463

[104] Huang Z Ma L Huang C Li Q Nice EC. Proteomic Profiling of Human Plasma for Cancer Biomarker Discovery. Proteomics (2017) 17. doi: 10.1002/pmic.201600240 27550791

[105] Yuan H Yan M Zhang G Liu W Deng C Liao G. CancerSEA: A Cancer Single-Cell State Atlas. Nucleic Acids Res (2019) 47:D900–8. doi: 10.1093/nar/gky939 30329142 PMC6324047

[106] Buisson R Langenbucher A Bowen D Kwan EE Benes CH Zou L. Passenger Hotspot Mutations in Cancer Driven by APOBEC3A and Mesoscale Genomic Features. Science (2019) 364. doi: 10.1126/science.aaw2872 PMC6731024 31249028

[107] Seton-Rogers S. Passengers Masquerading as Cancer Drivers, Nature Reviews. Cancer (2019) 19:485. doi: 10.1038/s41568-019-0184-y 31337868

[108] Blokzijl F de Ligt J Jager M Sasselli V Roerink S Sasaki N. Tissue-Specific Mutation Accumulation in Human Adult Stem Cells During Life. Nature (2016) 538:260–4. doi: 10.1038/nature19768 PMC5536223 27698416

[109] Martincorena I Roshan A Gerstung M Ellis P Van Loo P McLaren S. Tumor Evolution. High Burden and Pervasive Positive Selection of Somatic Mutations in Normal Human Skin. Science (2015) 348:880–6. doi: 10.1126/science.aaa6806 PMC4471149 25999502

[110] Chappell G Pogribny IP Guyton KZ Rusyn I. Epigenetic Alterations Induced by Genotoxic Occupational and Environmental Human Chemical Carcinogens: A Systematic Literature Review, Mutation Research. Rev Mutat Res (2016) 768:27–45. doi: 10.1016/j.mrrev.2016.03.004 PMC4884606 27234561

[111] Birkett N Al-Zoughool M Bird M Baan RA Zielinski J Krewski D. Overview of Biological Mechanisms of Human Carcinogens, Journal of Toxicology and Environmental Health. Part B Crit Rev (2019) 22:288–359. doi: 10.1080/10937404.2019.1643539 31631808

[112] Smith MT Guyton KZ Gibbons CF Fritz JM Portier CJ Rusyn I. Key Characteristics of Carcinogens as a Basis for Organizing Data on Mechanisms of Carcinogenesis. Environ Health Perspect (2016) 124:713–21. doi: 10.1289/ehp.1509912 PMC4892922 26600562

[113] Muñoz-Espín D Cañamero M Maraver A Gómez-López G Contreras J Murillo-Cuesta S. Programmed Cell Senescence During Mammalian Embryonic Development. Cell (2013) 155:1104–18. doi: 10.1016/j.cell.2013.10.019 24238962

[114] Corsini NS Knoblich JA. Tracing Stem Cell Division in Adult Neurogenesis. Cell Stem Cell (2018) 22:143–5. doi: 10.1016/j.stem.2018.01.012 29451854

[115] Sánchez-Taltavull D. Optimal Architecture of Differentiation Cascades With Asymmetric and Symmetric Stem Cell Division. J Theor Biol (2016) 407:106–17. doi: 10.1016/j.jtbi.2016.07.02 27452530

[116] Murata M. Inflammation and Cancer. Environ Health Prev Med (2018) 23:50. doi: 10.1186/s12199-018-0740-1 30340457 PMC6195709

[117] Katoh M. Multi−layered Prevention and Treatment of Chronic Inflammation, Organ Fibrosis and Cancer Associated With Canonical WNT/β−Catenin Signaling Activation (Review). Int J Mol Med (2018) 42:713–25. doi: 10.3892/ijmm.2018.3689 PMC6034925 29786110

[118] Clifford GM Tully S Franceschi S. Carcinogenicity of Human Papillomavirus (HPV) Types in HIV-Positive Women: A Meta-Analysis From HPV Infection to Cervical Cancer. Clin Infect Dis Off Publ Infect Dis Soc Am (2017) 64:1228–35. doi: 10.1093/cid/cix135 PMC5399941 28199532

[119] Gaillard H García-Muse T Aguilera A. Replication Stress and Cancer, Nature Reviews. Cancer (2015) 15:276–89. doi: 10.1038/nrc3916 25907220

[120] Jyoti S Tandon S. Chemical and Physical Factors Influencing the Dynamics of Differentiation in Embryonic Stem Cells. Curr Stem Cell Res Ther (2015) 10:477–91. doi: 10.2174/1574888x10666150416113055 25882851

[121] Clause KC Liu LJ Tobita K. Directed Stem Cell Differentiation: The Role of Physical Forces. Cell Commun Adhes (2010) 17:48–54. doi: 10.3109/15419061.2010.492535 20560867 PMC3285265

[122] Bhagat TD Chen S Bartenstein M Barlowe AT Von Ahrens D Choudhary GS. Epigenetically Aberrant Stroma in MDS Propagates Disease *via* Wnt/β-Catenin Activation. Cancer Res (2017) 77:4846–57. doi: 10.1158/0008-5472.Can-17-0282 PMC5600853 28684528

[123] Roulot A Héquet D Guinebretière JM Vincent-Salomon A Lerebours F Dubot C. Tumoral Heterogeneity of Breast Cancer. Ann Biol Clin (2016) 74:653–60. doi: 10.1684/abc.2016.1192 27848916

[124] Flavahan WA Gaskell E Bernstein BE. Epigenetic Plasticity and the Hallmarks of Cancer. Science (2017) 357. doi: 10.1126/science.aal2380 PMC5940341 28729483

[125] Tang J Luo Y Wu G. A Glycolysis-Related Gene Expression Signature in Predicting Recurrence of Breast Cancer. Aging (2020) 12:24983–94. doi: 10.18632/aging.103806 PMC7803557 33201835

[126] Xi Y Fowdur M Liu Y Wu H He M Zhao J. Differential Expression and Bioinformatics Analysis of circRNA in Osteosarcoma. Biosci Rep (2019) 39. doi: 10.1042/bsr20181514 PMC6522716 31036604

[127] Sell S. On the Stem Cell Origin of Cancer. Am J Pathol (2010) 176:2584–494. doi: 10.2353/ajpath.2010.091064 PMC2877820 20431026

[128] Eun K Ham SW Kim H. Cancer Stem Cell Heterogeneity: Origin and New Perspectives on CSC Targeting. BMB Rep (2017) 50:117–25. doi: 10.5483/bmbrep.2017.50.3.222 PMC5422023 27998397

[129] Prasetyanti PR Medema JP. Intra-Tumor Heterogeneity From a Cancer Stem Cell Perspective. Mol Cancer (2017) 16:41. doi: 10.1186/s12943-017-0600-4 28209166 PMC5314464

[130] Chaffer CL Weinberg RA. How Does Multistep Tumorigenesis Really Proceed? Cancer Discov (2015) 5:22–4. doi: 10.1158/2159-8290.Cd-14-0788 PMC4295623 25583800

[131] Hanahan D Weinberg RA. Hallmarks of Cancer: The Next Generation. Cell (2011) 144:646–74. doi: 10.1016/j.cell.2011.02.013 21376230

[132] Fang X Zhong G Wang Y Lin Z Lin R Yao T. Low GAS5 Expression may Predict Poor Survival and Cisplatin Resistance in Cervical Cancer. Cell Death Dis (2020) 11:531. doi: 10.1038/s41419-020-2735-2 32661236 PMC7359315

[133] Sheng W LaFleur MW Nguyen TH Chen S Chakravarthy A Conway JR. LSD1 Ablation Stimulates Anti-Tumor Immunity and Enables Checkpoint Blockade. Cell (2018) 174:549–63.e519. doi: 10.1016/j.cell.2018.05.052 29937226 PMC6063761

[134] Qin Y Vasilatos SN Chen L Wu H Cao Z Fu Y. Inhibition of Histone Lysine-Specific Demethylase 1 Elicits Breast Tumor Immunity and Enhances Antitumor Efficacy of Immune Checkpoint Blockade. Oncogene (2019) 38:390–405. doi: 10.1038/s41388-018-0451-5 30111819 PMC6336685

[135] Saini A Gallo JM. Epigenetic Instability may Alter Cell State Transitions and Anticancer Drug Resistance. PloS Comput Biol (2021) 17:e1009307. doi: 10.1371/journal.pcbi.1009307 34424912 PMC8412323

[136] Ishay-Ronen D Diepenbruck M Kalathur RKR Sugiyama N Tiede S Ivanek R. Gain Fat-Lose Metastasis: Converting Invasive Breast Cancer Cells Into Adipocytes Inhibits Cancer Metastasis. Cancer Cell (2019) 35:17–32.e16. doi: 10.1016/j.ccell.2018.12.002 30645973

[137] Ishay-Ronen D Christofori G. Targeting Cancer Cell Metastasis by Converting Cancer Cells Into Fat. Cancer Res (2019) 79:5471–5. doi: 10.1158/0008-5472.Can-19-1242 31331908

